# Controllable large positive and negative Goos–Hänchen shifts with a double-Lambda atomic system

**DOI:** 10.1038/s41598-023-30632-w

**Published:** 2023-03-07

**Authors:** Anas Othman, Saeed Asiri, M. Al-Amri

**Affiliations:** 1 Department of Physics, Faculty of Science, Taibah University, Al Madinah Al Munawwarah, Saudi Arabia; 2 Institute of Quantum Technologies and Advanced Computing, KACST, Riyadh, 11442 Saudi Arabia; 3 NCQOQI, KACST, Riyadh, 11442 Saudi Arabia

**Keywords:** Quantum physics, Quantum optics, Physics

## Abstract

We study the Goos–Hänchen shift (GHS) of a reflected light beam from a cavity containing a double-$\Lambda$ atomic medium that is bounded by two glass slabs. Applying both coherent and incoherent fields to the atomic medium leads to positive and negative controllability of GHS. For some specific values of the parameters of the system, the amplitude of the GHS becomes large, namely, in the order of $\sim 10^{3}$ times the wavelength of the incident light beam. These large shifts are found at more than one angle of incidence with a wide range of parameters of the atomic medium.

## Introduction

Goos–Hänchen shift (GHS) is a phenomena, which occurs when a light beam is incident on a medium with a refractive index smaller than that of the medium of incidence. For an angle of incidence greater than the critical angle, the incident beam penetrates for some distance inside the second medium^1–6^ and reflects back to the first (incident) medium, in which the reflected beam is laterally shifted at the interface from the point at which the incident beam entered the second medium. This lateral displacement is named Goos–Hänchen shift after its experimental demonstration in 1947 by Goos and Hänchen^7,8^. Several theoretical proposals have been suggested to calculate the GHS such as the stationary phase method , which was developed by Artmann^9^. Another method based on the concept of energy conservation was introduced by Renard to theoretically calculate the GHS^10^.

Many structures and designs with different materials have been proposed to measure and control the GHS. For example, studying GHS in low absorbing media^11–13^ and in epsilon-near-zero slab^14,15^. Also, in different arrangements of defective and normal photonic crystals^16–18^. Further examples of the investigation of GHS include using two layers of different artificial media^19–21^, a cavity containing colloidal ferrofluids^22^ and graphene layers^23,24^ are all reported. More recently, GHS with an amplitude reaching four times of the wavelength of the incident light is obtained in a structure containing a periodic grating layer^25,26^. In addition to all of the previous examples, GHS was also observed experimentally for a transmitted beam in one-dimensional photonic crystal slabs^27^.

On the other hand, various atomic media where the optical properties of these media can be modified by some external parameters such as coherent fields were proposed and applied for different purposes^28–33^. The use of such atomic media to manipulate and control the GHS^34–38^ has been suggested. In^34^, a driven two-level system is used in a three layers cavity to coherently control the GHS. In^37,39^, the GHS is studied using the same cavity structure and containing a $\Lambda$ atomic scheme, where positive and negative lateral shifts were reported. Moreover, different four-level atomic structures^40–42^ including the double-$\Lambda$ atomic system^43,44^ are studied along with different techniques.

In this report, we show that the double-$\Lambda$ atomic system, which has two probe interactions can be used to produce large GHS in the order of $10^3 \lambda$. The double-$\Lambda$ scheme has relatively large controllable dispersion feature greater than the $\Lambda$ atomic scheme with limited absorption^45^. This great controllability makes the double-$\Lambda$ scheme an excellent candidate to produce very large GHS. Therefore, we study the effect of different parameters on the GHS in a cavity containing three layers where the middle layer is filled by the double-$\Lambda$ atoms.

## Results

### Model

**Figure 1:**
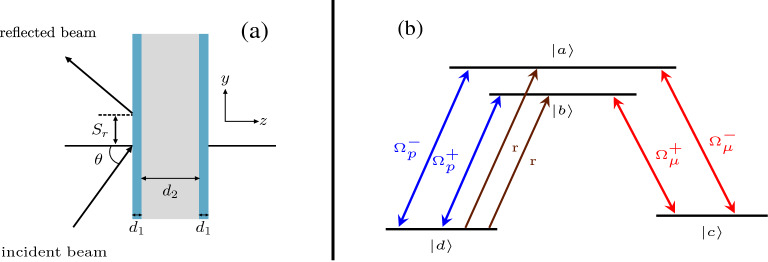
(**a**) Configuration of the three layers cavity, which consists of two glass slabs having the same thickness $d_1$ surrounding an intracavity of thickness $d_2$. A light beam is incident on the cavity with an angle of incidence $\theta$ and the reflected beam is laterally shifted on the *y*-axis. This lateral shift $S_r$ is known as the Goos–Hänchen Shift (GHS). (**b**) The double-$\Lambda$ atomic scheme, which is placed in the intracavity to control the GHS.

We consider a TE-polarized light field with a frequency $\omega _{p}$ is incident from vacuum with an angle $\theta$ on a cavity consisting of three layers of nonmagnetic materials. The first and last layers are identical and have a thickness $d_1$, while the middle layer has a thickness $d_2$ as shown in Fig. 1a. The electric permittivity of the edge and intracavity layers are $\epsilon _1$ and $\epsilon _2$, respectively. The double-$\Lambda$ atomic medium is placed in the second layer. The atomic system as shown in Fig. 1b has four levels ($|a\rangle$, $|b\rangle$, $|c\rangle$, and $|d\rangle$) where the transitions $|a\rangle$ $\leftrightarrow$ $|d\rangle$ and $|b\rangle$ $\leftrightarrow$ $|d\rangle$ are coupled by two probe fields with Rabi frequencies $\Omega _p^-$ and $\Omega _p^+$, respectively. Two strong coherent fields are driving the transitions $|a\rangle$ $\leftrightarrow$ $|c\rangle$ and $|b\rangle$ $\leftrightarrow$ $|c\rangle$ with Rabi frequencies $\Omega _\mu ^-$ and $\Omega _\mu ^+$, respectively. Also, the system is pumped by two incoherent fields from the state $|d\rangle$ to $|a \rangle$ and $|b \rangle$ with the same rate *r*. The double-$\Lambda$ system exists for example in rubidium and sodium^46,47^. We choose the D$_{2}$ transition in ${}^{85}$Rb where the states $|a\rangle$ and $|b\rangle$ correspond to the hyperfine levels with $F=4, m_{F} = 0$ and $F=3, m_{F} = 0$, respectively. The lower levels $|c \rangle$ and $|d \rangle$ correspond to the hyperfine level $F=3$ with magnetic sublevels $m_{F} = +1$ and $m_{F} = -1$, respectively. Therefore, right and left circularly polarized fields ($\sigma ^{\pm }$) are used for both the probe and driving fields. All different fields are assumed to be homogeneous through the whole cavity.

The Hamiltonian of the double-$\Lambda$ atomic system^45^ in the dipole and rotating wave approximations is written as

1 $$\begin{aligned} \begin{aligned} H&= \hbar \bigg [ \omega _a |a\rangle \langle a| + \omega _b |b\rangle \langle b| + \omega _c |c\rangle \langle c| + \omega _d |d\rangle \langle d| - \dfrac{\Omega _p^-}{2} e^{i \Delta _{1} t} |a\rangle \langle d| - \dfrac{\Omega _p^+}{2} e^{i \Delta _{2} t} |b\rangle \langle d| - \dfrac{\Omega _\mu ^+}{2} e^{i \Delta _{3} t} |b\rangle \langle c| \\&\quad - \dfrac{\Omega _\mu ^-}{2} e^{i \Delta _{4} t} |a\rangle \langle c| +H.c. \bigg ], \end{aligned} \end{aligned}$$

where $\omega _{a}, \omega _{b}, \omega _{c},$ and $\omega _{d}$ are the frequencies of the energy levels $|a\rangle , |b\rangle , |c\rangle ,$ and $|d\rangle$, respectively. The Rabi frequencies of the two probe fields are $\Omega _p^-$ and $\Omega _p^+$, whereas the Rabi frequencies of the driving fields are $\Omega _\mu ^-$ and $\Omega _\mu ^+$. The detunings in Eq. (1) are defined such that $\Delta _{1} = \omega _{ad} - \omega _{p}$, $\Delta _{2} = \omega _{bd} - \omega _{p}$, $\Delta _{3} = \omega _{bc} - \omega _{\mu }$, and $\Delta _{4} = \omega _{ac} - \omega _{\mu }$, where we assumed that the two probe fields have the same frequency $\omega _{p}$, and the two driving fields have the same frequency $\omega _\mu$. The equations of motion for the density matrix elements can be derived using the master equation^45,48^ along with the Hamiltonian Eq. (1). These equations of motion can be solved to the first order at steady state when considering weak probe of the system. The permittivity of the middle layer $\varepsilon _{2}$ is defined in terms of the susceptibility of the atomic system as $\varepsilon _{2} = 1+ \chi$. The dielectric susceptibility of the system^45^ has two parts $\chi _{ad}$ and $\chi _{bd}$, which come from the double probe interactions with the atomic medium. Therefore, the susceptibility is expressed as $\chi = \chi _{ad} + \chi _{bd}$ in which these two parts are given by

2 $$\begin{aligned} \chi _{bd}= \frac{i}{D_{bd}+\frac{|\Omega _\mu ^+|^2}{4D_{cd}}} \left[ \mathscr{C}\left( \frac{ |\Omega _\mu ^+|^2}{4 D_{cd} D_{bc}^* } P_{bc} - P_{bd}\right) - \mathscr{B} \left( \frac{ \Omega _\mu ^+ \Omega _\mu ^{-*}}{4 D_{cd} D_{ac}^*} P_{ca}\right) \right] , \end{aligned}$$

and

3 $$\begin{aligned} \chi _{ad}=\frac{i}{D_{ad}+\frac{|\Omega _\mu ^-|^2}{4D_{cd}}} \left[ \mathscr{B} \left( \frac{\Omega _\mu ^- \Omega _\mu ^{+*}}{4 D_{cd} D_{bc}^*} P_{bc}\right) -\mathscr{A} \left( \frac{| \Omega _\mu ^-|^2}{4 D_{cd} D_{ac}^*} P_{ca}+ P_{ad} \right) \right] , \end{aligned}$$

where $$D_{bd}=\gamma _{bd}-i(\Delta +\omega _{ab}/2)$$, $$D_{ad}=\gamma _{ad}-i(\Delta -\omega _{ab}/2)$$, $$D_{cd}=\gamma _{cd}-i(\Delta _\mu +\Delta )$$, $$D_{bc}=\gamma _{bc}+i(\Delta _\mu -\omega _{ab}/2)$$ and $$D_{ac}=\gamma _{ac}+i(\Delta _\mu +\omega _{ab}/2)$$.

The parameter $P_{ij} = \rho ^{(0)}_{ii}- \rho ^{(0)}_{jj}$, is the population difference between the states $|i\rangle$ and $|j\rangle$ where $i, j \in (a, b, c, d)$. The expressions of these populations are given as^45^

4a $$\begin{aligned} \rho ^{(0)}_{aa}&= r \dfrac{R_{a} (\gamma _{b} + \gamma _{B}) + \gamma _{B} R_{a}+ \gamma _{b} R_{b} + 2 R_{a} R_{b}}{a_{1} R_{a} + a_{2} R_{b} + a_{3} R_{a} R_{b} + a_{4}}, \end{aligned}$$

4b $$\begin{aligned} \rho ^{(0)}_{bb}&= r \dfrac{R_{b} (\gamma _{a} + \gamma _{A}) + \gamma _{a} R_{a}+ \gamma _{A} R_{b} + 2 R_{a} R_{b}}{a_{1} R_{a} + a_{2} R_{b} + a_{3} R_{a} R_{b} + a_{4}}, \end{aligned}$$

4c $$\begin{aligned} \rho ^{(0)}_{dd}&= \dfrac{\gamma _{a} \rho _{aa} + \gamma _{b} \rho _{bb}}{2 r}, \end{aligned}$$

4d $$\begin{aligned} \rho ^{(0)}_{cc}&= 1 - \rho ^{(0)}_{aa} -\rho ^{(0)}_{bb} -\rho ^{(0)}_{cc}. \end{aligned}$$

The detailed expressions of the rest the parameters $a_{1}$, $a_{2}$, $a_{3}$, $a_{4}$, $R_{a}$, and $R_{b}$ can be found in^45^. The decay rates are denoted by $\gamma _i$, and $\gamma _{ij} =(\gamma _i+\gamma _j)/2$, is the average of the decay rates of the states $|i \rangle$ and $|j \rangle$. The values of the decay rates are $\gamma _{a} = \gamma _{b} = 0.7 \gamma$, $\gamma _{A} = \gamma _{B} = 0.2 \gamma$, $\gamma _{ab} = \gamma _{cd} = 0$, and $\gamma _{ac} = \gamma _{bc} = \gamma _{ad} = \gamma _{bd} = (\gamma _{a} + \gamma _{A})/2 = 0.5 \gamma$, where $\gamma = 10$ MHz. The parameters $\Delta$ and $\Delta _\mu$ are defined as $\Delta =\omega _{p} - {W_{p}}$ and $\Delta _\mu = {W_{\mu }} -\omega _{\mu }$, where ${W_{p}} = (\omega _{ad}+\omega _{bd})/2$, ${W_{\mu }} = (\omega _{ac}+\omega _{bc})/2$, and $\omega _{ij} = \omega _i-\omega _j$ is the energy difference between the two states $|i \rangle$ and $|j \rangle$. $\mathscr{A}, \mathscr{B}, \mathscr{C}$ are the density parameters. Also, $\Omega _\mu ^+ = \Omega _\mu ^-/\alpha$, where $\alpha$ is the ratio between the two driving fields.

### The GH shift

The GHS of the reflected TE-polarized light field $S_{r}$ can be calculated using the result of the stationary phase theory^9^ , which is given by

5 $$\begin{aligned} S_{r} = - \frac{d \phi _r}{d k_{y}}, \end{aligned}$$

where $k_{y} = k {\sin } \theta$ is the parallel component of the wavevector, $k = \omega _{p} /c$ where *c* is the speed of light in vacuum. The function $\phi _{r}$ represents the phase shift , which corresponds to the reflected field. The phase shift of the reflected TE-polarized field is directly related to the reflection coefficient $r^{{\textrm{TE}}}$ via $\phi _{r} = {\tan }^{-1} \big [ {\textrm{Im}}(r^{{\textrm{TE}}})/{\textrm{Re}}(r^{{\textrm{TE}}}) \big ]$.

We calculate the reflection coefficient $r^{{\textrm{TE}}}$ of the three layers cavity for the TE-polarized field using the standard characteristic matrix approach^49,50^, which enables connecting the field through the layers of the cavity. Following the same approach as, for example in^34,37^, the reflection coefficient for the TE-polarized field $r^{{\textrm{TE}}}$ is given as

6 $$\begin{aligned} r^{{\textrm{TE}}}(\theta ,\omega _{p})=\frac{\cos \theta (X^{{\textrm{TE}}}_{22}-X^{{\textrm{TE}}}_{11})-({\cos ^{2}\theta } X^{{\textrm{TE}}}_{12}-X^{{\textrm{TE}}}_{21})}{\cos \theta (X^{{\textrm{TE}}}_{22}+X^{{\textrm{TE}}}_{11})-({\cos ^{2}\theta } X^{{\textrm{TE}}}_{12}+X^{{\textrm{TE}}}_{21})}, \end{aligned}$$

where ${X}^{{\textrm{TE}}}_{ij}$ is the matrix element of the total transfer matrix of the three layers cavity. The total transfer matrix for our configuration is given by

7 $$\begin{aligned} X^{{\textrm{TE}}}(\theta ,\omega _{p})=M^{{\textrm{TE}}}_{1}(\theta ,\omega _{p}, {d_1}) M^{{\textrm{TE}}}_{2}(\theta ,\omega _{p}, {d_2}) M^{{\textrm{TE}}}_{1}(\theta ,\omega _{p}, {d_1}). \end{aligned}$$

For any single layer, the transfer matrix can be calculated from

8 $$\begin{aligned} \ {M}^{{\textrm{TE}}}_{j}(\theta ,\omega _{p},d_{j})= & {} \begin{pmatrix} \cos [n_{j}k\cos (\theta _{j})d_{j}] &{} i\sin [n_{j}k\cos (\theta _{j})d_{j}]/(n_{j}\cos (\theta _{j}) \\ i n_{j}\cos (\theta _{j}) {\sin }[n_{j}k\cos (\theta _{j})d_{j}] &{} {\cos }[n_{j}k\cos (\theta _{j})d_{j}] \\ \end{pmatrix}, \end{aligned}$$

where $\sin \theta _{j}=\sin \theta /n_{j}$, $k=\omega _{p}/c$ is the wave number of the incident probe field in vacuum with probe frequency $\omega _p$, while $n_{j}$ is the refractive index of the *j*-th layer in the cavity, and $d_j$ is the thickness of the jth layer.

The parameters in our configuration can be selected to be similar to most of the articles applying the same three layers cavity. The thickness of the layers are $d_1 = 0.2 \;\, {\mu \textrm{m}}$, $d_2 = 5 \,\, {\mu \textrm{m}}$, and the permittivity of the edge layers is $\epsilon _1 =2.22$. Next, the parameters of the double-$\Lambda$ atomic medium are^45^ as follows: $\omega _{ab} = 12.1 \gamma$, $W = 2 \pi \times 300$ THz, $\Delta = -5 \gamma$, $\Delta _\mu = 0$, $\mathscr{A} = 1.1 \gamma$, $\mathscr{B} = 1.05 \gamma$, and $\mathscr{C} =\gamma$, where $\gamma =10$ MHz. The free parameters, which will be studied are $\Omega _\mu$, *r*, and $\theta$, where $\Omega _\mu ^- = \Omega _\mu ^+ = \Omega _\mu$ and $\alpha =1$.

**Figure 2:**
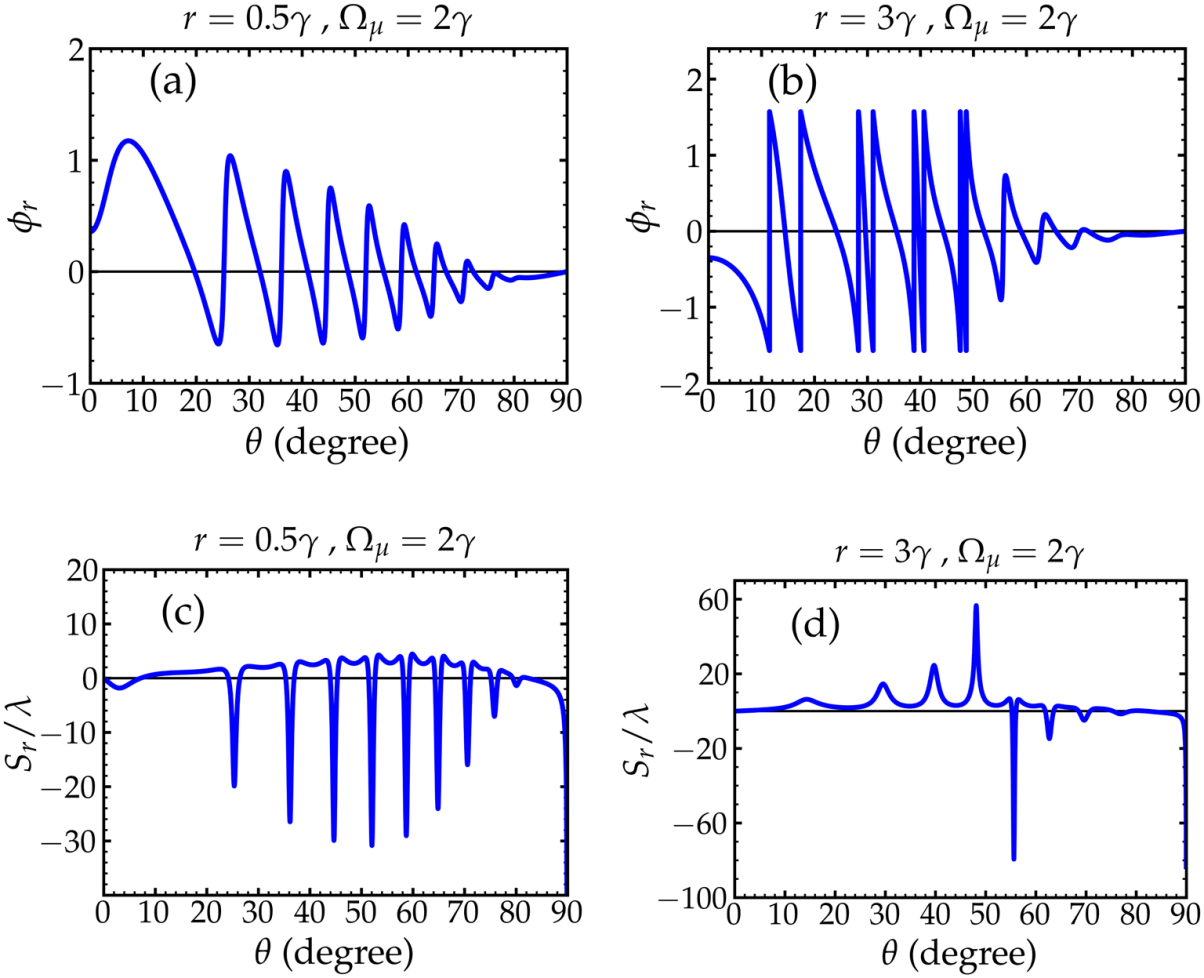
(**a**) and (**b**) shows the relative phase of the reflected beam versus the angle of incidence $\theta$. (**c**) and (**d**) shows the dependence of the GHS of the reflected light beam on the angle of incidence $\theta$. The pumping rate values are $r = 0.5 \gamma$ in (**a**) and (**c**), while $r = 3 \gamma$ in (**b**) and (**d**). The driving field $\Omega _{\mu } = 2 \gamma$ in (**a**)–(**d**). The amplitude of the GHS becomes large at angles of incidence where sharp phase changes occur. Other parameters are shown in the text.

Next, we proceed with the calculations of the GHS. To have a glance over the capability of our system to control the GHS, we plot the GHS of the reflected beam versus the angle of incidence $\theta$ from 0 to $\pi /2$ for some selected parameters of the atomic medium. We see in Fig. 2 that both the amplitude and direction of the GHS can be changed when changing *r*. Needless to mention that our system is tuned and controlled remotely by simply manipulating the values of the pump *r* and Rabi frequency of the driving fields $\Omega _\mu$ yielding a significant change in the behavior of the GHS, while the cavity structure is kept intact.

In the following, we study the dependence of the lateral shift of the reflected beam on the external parameters *r* and $\Omega _\mu$. Our purpose is to see the behavior of the GHS by only changing the parameters of the atomic medium, namely *r* and $\Omega _\mu$, and without changing the structure of the cavity. We also can find out which values of *r* and $\Omega _\mu$ can produce large GHS.

### The effect of *r*

**Figure 3:**
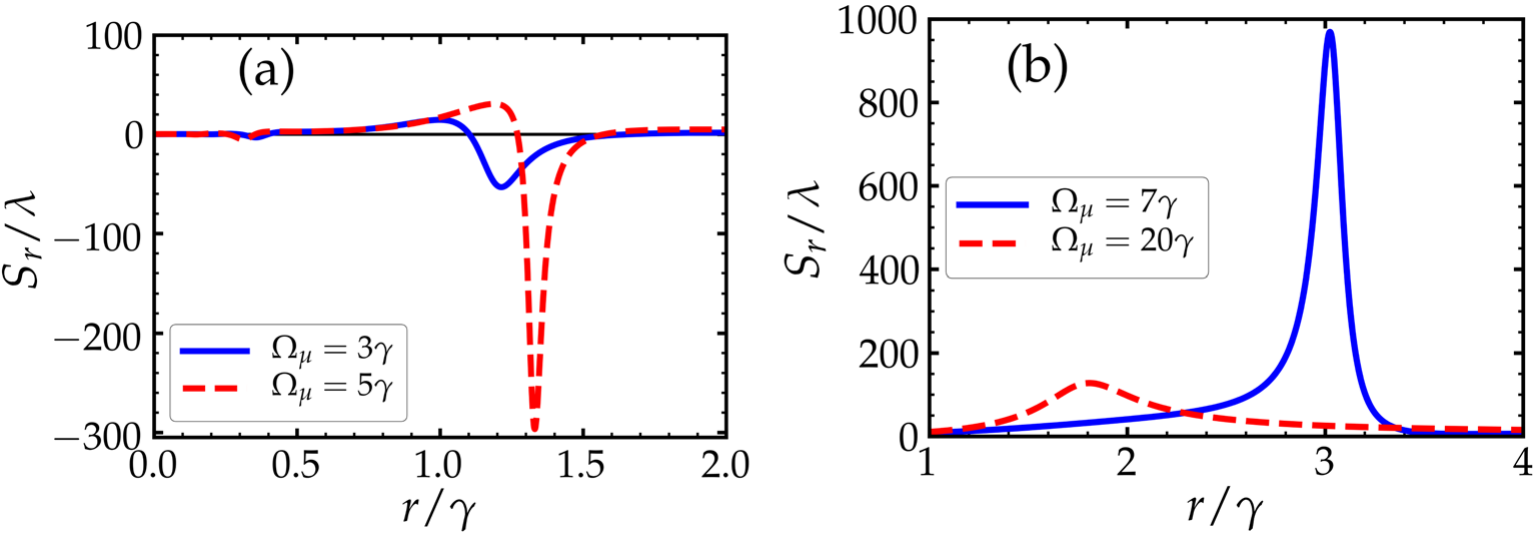
The GHS of the reflected light field $S_r$ verses the pumping rate *r* for different values of driving fields $\Omega _\mu$. The driving field values in (**a**) are $\Omega _{\mu }=3 \gamma$ (solid) and $\Omega _{\mu }=5 \gamma$ (dashed). Similarly, $\Omega _{\mu }=7 \gamma$ (solid) and $\Omega _{\mu }=20 \gamma$ (dashed) in (**b**). Other parameters are shown in the text.

We study the effect of the pumping rate *r* on the GHS while the driving fields are fixed. In Fig. 3, we plot the GHS of the reflected light beam $S_r$ versus *r* for different values of $\Omega _\mu$, while the angle of incidence is assumed to be $\theta = {62^{\circ }}$. The GHS in Fig. 3 can be positive or negative for the selected values of $\Omega _\mu$. In Fig. 3a, it is noticed that around some specific values of the pumping rate *r*, the GHS is large in comparison to the wavelength of the incident light beam, i.e. , in the order of $10^{2} \lambda$ when $\Omega _\mu = 5 \gamma$. When $\Omega _\mu = 7 \gamma$, large positive GHS in the order of nearly $10^{3} \lambda$ occurs at $r \approx 3 \gamma$ as seen in Fig. 3b.

### The effect of $\Omega _\mu$

**Figure 4:**
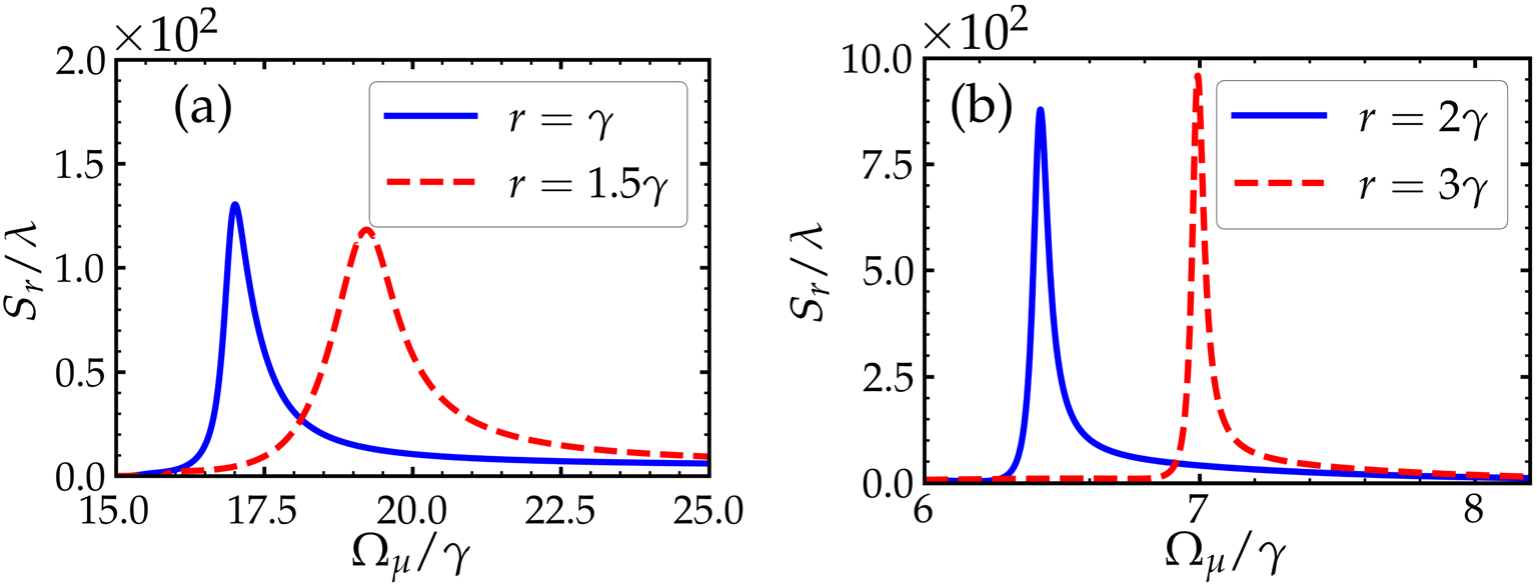
The dependnce of the GHS on the driving fields $\Omega _\mu$ for different values of pumping rates *r*. Other parameters are shown in the text.

In this subsection, we explore the dependence of the GHS of the reflected beam on the Rabi frequency of the driving fields $\Omega _\mu$. From the previous study (Sec. III. A), we obtained large GHS at certain values of *r*. Figure 4 shows the dependence of the GHS on $\Omega _\mu$, where large negative and positive GHS occur over some range of $\Omega _\mu$.

In Fig. 4a, we plot the GHS with $\Omega _\mu$ for two different values of *r*. We observe large positive shifts in the order of $~ 10^2 \lambda$ over a relatively wide range of $\Omega _\mu$. This indicates it is flexible to choose the value of $\Omega _\mu$ that produces a large positive GHS in this situation.

In Fig. 4b, we see that large shifts are achieved at different points of $\Omega _\mu$ as *r* is modified. For example, when $r = 3 \gamma$, we observe a large positive GHS, i.e. , $S_{r} \approx - 10^{3} \lambda$ at $\Omega _\mu \approx 7 \gamma$. In fact, these large shifts are continuous in the selected range of the values of *r*. Therefore, this suggests that we can pick a pair (*r*, $\Omega _\mu$) that produces large shifts in the orders of $~ 10^3 \lambda$.

### Other angles

So far, the analysis of the GHS have been carried out when the angle of incidence is $\theta = {62^{\circ }}$. It should be pointed out that not all angles under our selected parameters can necessarily produce large shifts. Here, we show that large positive or negative GHS at other selected values of the angle of incidence can still be observed.

**Figure 5:**
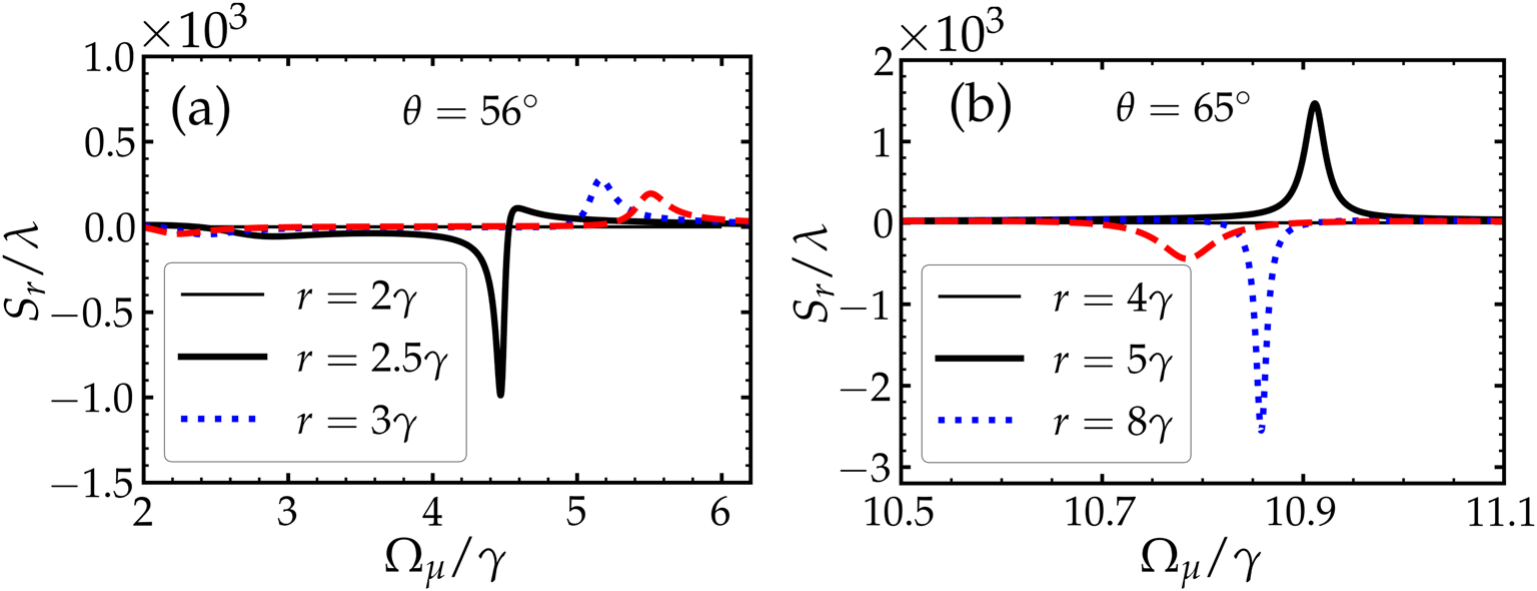
The GHS of the reflected beam against the driving field $\Omega _\mu$ for different values of the pumping rate *r*. The angles of incidence in (**a**) and (**b**) are $\theta ={56^{\circ }}$ and $\theta = {65^{\circ }}$, respectively. Other parameters are shown in the text.

In Fig. 5, we see that the GHS of the reflected beam reaches values of order $10^3 \lambda$ under specific values of the pair (*r*, $\Omega _\mu$). In all the reported results for the selected angles here, we observe large positive and negative GHS as the value of *r* is modified. For example, in Fig. 5a where the angle of incidence is $\theta = {56^{\circ }}$, the GHS is reaching an order of $10^3 \lambda$, which is relatively large shift where that occurs at a wide range of $\Omega _\mu$. Similarly in Fig. 5b where the angle of incidence is assumed to be $\theta = {65^{\circ }}$, large positive and negative GHS are observed for some specific values of *r* for a small range of $\Omega _\mu$. Thus, for each angle, to find a large GHS, one needs to perform some kind of an optimization in order to find the suitable value of the pair (*r*, $\Omega _\mu$) at which a large shift can occur.

### Incident Gaussian beam

All the previous results of the GHS are obtained using the expression derived by Artmann Eq. (5)^6,9^. Artmann derived this result to calculate the GHS assuming that the incident beam is a plane wave. When measuring the GHS experimentally, one would usually consider a laser beam, which has a Gaussian profile. As shown in^34^, we examine the validity of Artmann’s expression considering that the incident light in our system is a Gaussian beam, which can be written as

9 $$\begin{aligned} E_{x}^{i}(y, z=0) = \dfrac{1}{\sqrt{2 \pi }} \int B(k_{y}) e^{i k_{y} y} dk_{y}. \end{aligned}$$

Similarly, the reflected light beam at the interface is given by

10 $$\begin{aligned} E_{x}^{r}(y, z=0) = \dfrac{1}{\sqrt{2 \pi }} \int r^{{\textrm{TE}}} (k_{y}, \omega _{p}) B(k_{y}) e^{i k_{y} y} dk_{y}. \end{aligned}$$

**Figure 6:**
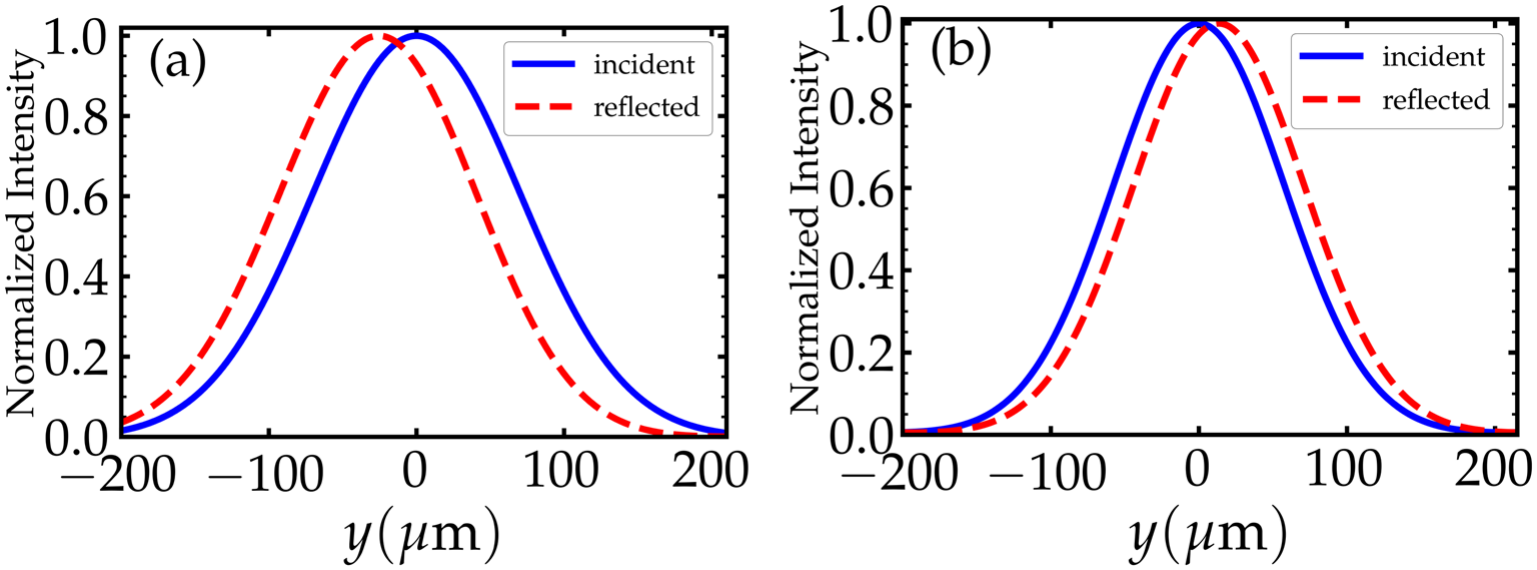
The normalized intensity distribution of the incident (solid) and reflected (dashed) light beams with $W = 100 \lambda$. The angle of incidence in (**a**) is $\theta = {44.6^{\circ }}$ with $r=0.5 \gamma$ and $\Omega _{\mu }=2 \gamma$. In (**b**), $\theta ={30^{\circ }}$ with $r= 3 \gamma$ and $\Omega _{\mu }=2 \gamma$. Other parameters are shown in the text.

Here $B(k_{y})$ is the angular spectrum distribution of the Gaussain beam , which is given by

11 $$\begin{aligned} B(k_{y}) = \dfrac{W_{y}}{\sqrt{2}} \; exp \Big [ \dfrac{W_{y}^2 (k_{y} - k_{y0})^{2}}{4} \Big ], \end{aligned}$$

with $W_{y} = W/{\cos } \theta$ and $k_{y0} = k {\sin } \theta$, where *W* represents the half width of the Gaussian beam at the interface. The position of the maximum normalized intensity distribution of the incident and reflected beams at the interface ($z=0$) can be calculated by

12 $$\begin{aligned} \langle {y^{i/r}} \rangle =\frac{\int y \big | E_{x}^{i/r}(y, z=0) \big |^2 dy}{\int \big | E_{x}^{i/r}(y, z=0) \big |^2 dy}, \end{aligned}$$

where the superscripts *i* and *r* indicate the incident and reflected beams, respectively. The GHS in this situation is given by the difference between the positions of the maximum points of the intensity profile of the incident and reflected beams, i.e., $\langle {y^{r}} \rangle - \langle {y^{i}} \rangle$. We choose $W = 100 \lambda$ in the calculations of the GHS using Eq. (12). In Fig. 6a, $\langle {y^{r}} \rangle - \langle {y^{i}} \rangle \approx -28 \,\, {\mu \textrm{m}}$ and in Fig. 6b $\langle {y^{r}} \rangle - \langle {y^{i}} \rangle \approx 13 \,\, {\mu \textrm{m}}$. These results of the lateral shift agree with the results , which are calculated using the stationary phase approach shown in Figs. 2a, 3b, respectively. Hence, this method confirms the validity of Artmann’s formula Eq. (5) of the GHS.

## Discussion

We investigated the control of the GHS of the reflected light beam using a double-$\Lambda$ atomic medium placed inside a cavity bounded by two glass slabs. We showed that the GHS of the reflected beam can be controlled remotely by simply changing the values of the pump *r* and Rabi frequency of the driving fields $\Omega _\mu$, while the cavity structure is kept intact. We also found that our system is capable of producing very large GHS of orders $10^{3} \lambda$ at more than one angle of incidence.

## Data Availability

The datasets that support the plots in this paper are available from the corresponding author on reasonable request.
